# Purified α-Amylase from *Bacillus cereus* exhibits antibiofilm and antiquorum sensing activities against uropathogenic *Escherichia coli*, Downregulating *fimH*, and *papC* virulence genes: implications for urinary tract infections

**DOI:** 10.1186/s12866-024-03542-8

**Published:** 2024-11-27

**Authors:** Amal M. Abo-Kamar, Abd-El-Rahman A. Mustafa, Lamiaa A. Al-Madboly

**Affiliations:** Department of Microbiology and Immunology, Faculty of Pharmacy, Tanta University, Tanta, Egypt

**Keywords:** Biofilm, *E. Coli*, *B. cereus*, CLSM, QRT-PCR, MBIC, MBEC, *recA*, *fimH*, *papC* genes

## Abstract

**Background and aim:**

Pathogenic *Escherichia coli* is a known harmful microorganism that takes advantage of favorable conditions to cause various infections in healthcare settings, such as bloodstream infections related to catheters, as well as infections in the urinary and respiratory tracts. *E. coli* utilizes biofilm development as a means to enhance its virulence and pathogenicity. This work aims to investigate the antibiofilm activity of α-amylase enzyme against uropathogenic *E. coli* (UPEC) and its effect on biofilm-regulatory genes.

**Methodology:**

In this study, we evaluated the antibiofilm activity of α-amylase enzyme by spectrophotometric microtiter plate analysis and confocal laser scanning microscopy. Also, the antibacterial activity of the test enzyme was evaluated by measuring the MIC and MBC levels against UPEC. The quorum-quenching activity of α-amylase enzyme was assessed using a qRT-PCR to evaluate the impact on biofilm-regulatory genes.

**Results:**

Based on our results, purified α-amylase showed MIC and MBC levels ranged between 128 and 512 µg /ml against UPEC isolates using broth microdilution assay. Crystal violet assay revealed MBIC of 128 µg/ml and MBEC of 256 µg/ml for the purified α-amylase. When the biofilm was analyzed by confocal laser scanning microscope, our results showed inhibition of biofilm thickness (56%) and live/dead cell percentages (43/55%). Furthermore, analysis of the effect on the expression of biofilm-encoding genes showed downregulation of both *fimH* and *papC* genes by 57 and 25%, respectively, upon treatment of UPEC with ½ of the MIC (64 µg/ml).

**Conclusions:**

The results demonstrate that our purified α-amylase from *B. cereus* exhibits promising antibiofilm activities against UPEC at both phenotypic as well as genotypic levels. These findings suggest that this enzyme may serve as a natural effective tool for removing bacterial biofilms, potentially offering new therapeutic avenues for treating infections caused by UPEC.

## Introduction

*Escherichia coli* is a prevalent pathogen responsible for several illnesses beyond the intestines, such as urinary tract infections, newborn meningitis, Gram-negative bacteremia, and prostatitis. The incidence of these infections is mostly determined by the host’s immune system and bacterial factors. *E. coli* pathogenicity is mainly attributed to its virulence factors, which contribute to host colonization and invasion, tissue damage, promotion of inflammatory responses, immune evasion, and biofilm formation. Previous investigations have shown that genes responsible for iron absorption systems (*iron*,* iuc*) and adhesins (*fim*,* afaI*,* sfa*,* pap*) are considered virulence factors [1].

Pathogenic *E. coli* and other categories of coliform bacteria. Bacterial attachment to the host cell surface often initiates through the utilization of specialized bacterial adhesins that possess pili. Additionally, adhesins of pili are elongated structures that protrude from the surface of bacteria and enable bacteria to adhere to host cells [2]. Urinary tract infections (UTIs) are globally recognized as a significant health risk. Uropathogenic *E. coli* (UPEC) is the primary culprit behind urinary tract infections, both those obtained in the community and those acquired in a hospital setting. The capacity of bacteria to endure and proliferate within biofilms seems to be the paramount determinant in the development of disease and the lack of treatment success. The production of biofilms by UPEC is considered a crucial element in the extended survival of bacterial cells in the urinary system and is accountable for the inflammatory reaction linked to urinary tract infections. Moreover, the emergence of drug resistance in strains linked with biofilms can considerably augment the challenge of treatment [3].

Two prevalent UPEC adhesins including type I pili (fimH) and P pili. The *fim* gene cluster encodes a type I pili, composed of the core protein fimA connected to the auxiliary proteins fimF and fimG, as well as the adhesion protein fimH. UPEC expresses P pili, which is strongly associated with the bacteria’s capacity to inhabit the kidney and induce pyelonephritis. The *Pap* gene cluster consists of 11 genes encoding the core component of the pilus rod (PapA), papEF encoding the adapter subunit, and the adhesion terminal PapG and various adhesion-related proteins, including papG and papC [3, 4]. RecA protein of *E. coli* is involved in many biological processes such as: general genetic recombination, DNA damage repair, participates in the SOS system caused by DNA damage binding to the ssDNA chain, recA forms a spiral filament structure called activated recA, is also involved in the inhibition of cell division and mutagenesis, that is why it was chosen as the management reference gene in this study [5, 6].

Quorum sensing (QS) plays a central role in the host microbiome, allowing bacteria to synchronize their behavior when population composition changes. The effects of quorum-sensing signaling molecules (QSMs) on the expression of various genes, such as those involved in biofilm formation, antibiotic production, and virulence factors, can be obtained by measuring the extracellular concentrations of secreted autoinducer molecules. The QS system displays different signals, and different bacteria secrete different signal molecules that can be recognized by associated receptors. For example, *N-*acyl homoserine lactones (AHLs) are the most common autoinducers of Gram-negative bacteria. Several enzymes have been characterized that degrade bacterial AHLs, such as acylases, amylases, and lactonases. AHL lactonases have been found in Gram-negative and Gram-positive bacteria and mammals. AHL lactonase efficiently hydrolyzes the cellular lactone ring of AHL, thereby inhibiting QS-related functions such as biofilm, virulence factors, and infection as determined by quorum quenching (QQ) activity [7]. This study aimed to evaluate the antibiofilm activity of α-amylase against uropathogenic *E. coli*. Also, we aimed to investigate the effect of the test enzyme on the expression of the relevant quorum-sensing coding genes.

## Materials and methods

### Bacterial isolates and culture media

In our study, 25 uropathogenic *Escherichia coli* (UPEC) isolates were obtained from the Departement of Microbiology and Immunology (Faculty of Pharmacy, Tanta University, Tanta, Egypt) and stored in ultrafreezer at -80 °C to study the antibacterial activities of α-amylase enzyme. Also, the reference standard *E. coli* strain (ATCC 700928) was used as a positive control in the present study.

Commercial α-amylase from *Bacillus amyloliquefaciens* was purchased from Sigma Aldrich (Germany) to be used as an authentic sample for comparing the activities versus purified enzyme from *Bacillus cereus* which was previously extracted and purified *via* precipitation using organic solvent (chilled ethanol ) in order to evaluate the antibiofilm effect [8].

Trypton soy agar (TSA) and Trypton soy broth (TSB) were used as culture media for our studies.

## Biochemical identification of bacterial isolates

Following the manufacturer’s instructions, a densitometer (BioMérieux, France) was used to adjust the bacterial suspension to a 0.5 McFarland turbidity standard. One colony at a time was chosen from the nutrient agar slant and suspended in 2.5 mL of 0.45% sterile aqueous saline (pH 7.0). To identify Gram-negative (GN) bacteria, data was entered into the VITEK^®^2 7.01 software (Biomerieux, France) within 30 min of inoculation into the NF-64 card/GN cassette. A reference strain of *E. coli* (ATCC 8729) was included in the test for normalization of results. The software is based on 47 biochemical tests that measure resistance, enzymatic activities, carbon source utilization, and more. It took around 8 h for the findings of the identification to become available. The VITEK 2 is an automated microbiological system utilizing growth-based technology used for bacterial identification [9].

## Estimation of antibacterial activity of purified α-amylase by determination of MIC and MBC level

In this study, the MIC and MBC levels of α-amylase previously purified from *B. cereus* by organic solvent precipitation were determined against 25 bacterial isolates of UPEC using the broth microdilution method [10]. For normalization and comparison of results, a commercial α-amylase enzyme from *B. amyloliqufaciens* (Sigma Aldrich, Germany) was chosen as a reference standard. Each test isolate of *E. coli* was inoculated into TSB at a concentration of 0.5 McFarland, then 100µL of serially two-fold diluted purified enzyme at concentration ranges from 512 to 32 µg/mL was added to each bacterial suspension, then incubated along with positive and negative controls at 37 °C for 24 h for determination of MIC values. The positive control is that well in which bacterial growth is observed, however, the negative control is a well containing only the culture medium. The MIC was defined as the lowest concentration of enzyme that inhibits visible bacterial growth. Cultures with concentrations equal to or above the MIC were transferred to tryptone soya agar medium and re-incubated for 24 h to measure MBC levels, plates with no visible colonies means that this concentration kills 99.9% of the bacteria and were denoted as the MBC. If the same result was obtained in two repeated experiments, the values ​​were set as MIC and MBC values [11].

## Antibiofilm activity of purified α-amylase against UPEC

### Biofilm formation and inhibitory assay

This test aims to screen for biofilm-forming uropathogenic *E. coli* isolates. Their ability to form biofilms was assessed using both spectrophotometric microtiter plate reader. Briefly, an overnight culture of the test isolates was used to inoculate TSB (pH 7) that was adjusted to 0.5 McFarland (1.5 × 10^8^ CFU/mL), which was then poured (100 µL) into sterile microtiter plate wells. Following incubation of test plates at 37 °C, planktonic cells were removed from wells by washing three times with sterile PBS then plates were left to air-dry for 15 min at room temperature. Wells containing biofilms were then stained with filter-sterilized crystal violet dye (0.1%) and incubated for 10 min to ensure appropriate staining of biofilms within the wells that were then washed again with sterile distilled water to remove excess stain and allowed to dry for 20 min at room temperature. Finally, wells were washed with 50 µl of 99% absolute ethanol and the optical density was evaluated at 595 nm using a spectrophotometer (SHIMADZU, UV-2450, Japan). Positive control was a well showed biofilm formation without treatment however the negative control represented by a medium without culture. All experiments were performed in triplicate and the mean values were calculated. Biofilm inhibition percent was estimated using the following equation:

Percentage of biofilm inhibition = optical density of control sample – optical density of test sample/optical density of control sample *100 [8]. 

The cutoff OD value (ODc) was defined as three standard deviations above the mean OD of the negative control. Isolates were classified as follows:

OD ≤ ODc = not a biofilm producer (-),

ODc < OD ≤ 2×ODc = weak biofilm producer (+),

2×ODc < OD ≤ 4×ODc = moderate biofilm producer (++),

4×ODc < OD = strong biofilm producer (+++).

All tests were carried out three times and the results were averaged.

### Determination of MBIC and MBEC of purified and commercial α-amylases against UPEC

In this experiment, purified and commercial α-amylases were used to evaluate their antibiofilm activity by determining MBIC and MBEC values. Pure overnight cultures of *E. coli* were inoculated into TSB at a concentration of 0.5 McFarland (1 × 10^8^ CFU/mL), then 100µL were transferred to 96-well polystyrene microtiter plates and incubated. Moreover, the culture medium was changed every 48 h to maintain cell viability. The OD of the wells was measured at a wavelength of 610 nm using an ELISA microplate reader (PowerWaveX, Bio-Tek, USA) to determine the absorbance, which indicates the ability to form biofilm. Plates with bacterial biofilm were used as positive controls and plates with culture medium alone were used as negative controls. Aliquots of about 50µL of serially diluted purified and commercial amylase enzymes with initial concentrations of 400 µg/mL, were transferred and diluted in the microtiter plate wells then incubated for 48 h at 37 °C. After incubation, the supernatant was discarded and each well was washed three times with 200 µl of sterile distilled water to remove unattached cells and dried for 20 min, stained with crystal violet for 10 min at room temperature, and then washed three times with water sterile distilled water to remove excess stain. After that, the dye was then fixed by 95% absolute methanol and the absorbance was then measured using a spectrophotometer (Biotech Instruments, USA). The MBIC is considered the minimum biofilm-inhibiting enzyme concentration beyond which the microorganisms cannot produce more biofilm biomass in the well and the OD values of the test well do not increase further. Minimum biofilm eradication concentration (MBEC) is considered the lowest concentration that kills 98% of the biofilm formed. At the bottom of the treated well. The experiment was performed in triplicate and the mean was calculated [8].

### Analysis of biofilm thickness and live/dead cell percentage calculations by CLSM and Fiji software

Uropathogenic *E. coli* biofilms were pre-established in TSB media in eight-well chamber slides for 2 days at 37 °C to form mature biofilms. Preformed mature biofilm was treated with 140 µg/mL of commercial or purified α-amylases (slightly above their MBEC values) and then re-incubated for another 48 h to evaluate the ability to eradicate the test biofilm. After incubation, positive control samples without enzyme were stained with live 5 µl of 0.01 mg/ml fluorescent acridine orange AO dye (Sigma Aldrich, Germany), excitation wavelength 488 nm and emission wavelength 515 nm. For test wells treated with commercial and purified α-amylases were stained by a mixture of acridine orange and propidium iodide fluorescent dyes (Mix of 1:1 diluted with 0.9% NaCL (5 µl of 0.01 mg/ml propedium iodided; PI with an excitation wavelength of 488 nm and emission wavelength 630 nm) to visualize live and dead cells. Freshly prepared acridine orange and propidium iodide (Sigma Aldrich, Germany) dyes were prepared for visualization of live/dead cells according to the manufacturer’s instructions. After staining, the slide remained at room temperature (25 °C) and protected from light for 10 min, then examined by a laser scanning fluorescence microscope (Leica DMI8, Germany). The experiment was repeated three times and the average value was calculated. For AO, it is a cell-penetrating fluorescent dye that emits green fluorescence when combined with dsDNA, however, PI is a fluorescent dye that cannot cross cell membranes and is generally confined to living cells. Therefore, living cells emit a green color, while dead cells with damaged membranes emit a red color [8].

Percent of thickness inhibition = thickness of control sample (µm) - thickness of test sample (µm)/thickness of control sample (µm) *100 as previously reported [8].

### CLSM quantification of live/dead cells

Confocal laser scanning microscopic images were analyzed using Fiji software (ImageJ, US National Institute of Health, Maryland, USA) as it is an open-source tool and freely available. To determine the live/dead cell ratio, the percentage of viable bacterial was evaluated by calculating the number of pixels corresponding with the total bacteria in the image (green + red), then calculating the number of pixels corresponding with the dead (red) bacteria in the image, and finally subtracting to find the number of pixels corresponding with live (green) bacteria. The live bacteria were quantified as a percentage of the total bacteria in each image, and therefore the area of red and green bacteria is proportional to the number of red and green bacteria. The resulting images were processed using a segmentation algorithm (Fiji, ImageJ) capable of separating signals between background and sample to obtain proportionality between microbial numbers and fluorescence signals. Images were converted to 32-bit grayscale and then the threshold option was used to separate the region of interest (ROI) from the region not covered by biofilm (referred to as “background”). More specifically, we measure the volume occupied by the fluorescence signal recorded at each pixel with the value above a specified grayscale threshold to distinguish specific signals from background noise. The software calculated the volume occupied by living microorganisms by measuring the volume occupied by that fluorescence [9, 10].

The specific sequence of operations in FIJI was as follows: First, images from the confocal microscope (.czi) were dragged to the Fiji bar to open it, when the file opened, we moved to the Image bar selected colour then selected split channels to separate green cells from red cells, then find the brightest frame and select Image then adjust threshold then check default and click apply, check: black background, as a result, we will have black and white images, procedures repeated for red and green channels. After that, splited the separate cells that appears as one by going to Process > Binary > Watershed then from analyze menu we selected to analyze particles, leave the size value at 0-infinity and selected add to manager option and clicked OK, then we go to window menu and selected ROI manager to view all regions of interset and select them, then we go to plugins menu and select colour segmentation option which was downloaded before and select dark live, light live, dead colour fronts for each image by multi-point tool, then click run, the program then shows a table with area percentage for each image segmentation analysis, then by using excel sheet, we calculated the live/dead area percentage for each confocal image. To find out how many live cells there are, we followed these steps for the green channel. After that, Result windows displayed a table with the number of ROIs and different measurements that we could select in Analyze -> Set Measurements. We repeated particle analysis for red channels too and saved the results in excel sheet. To calculate the percentage of live cells, we divided the number of spots detected in the green channel by the total number of green and red cells in the image and multiply by 100%. The following equations was used to calculate the percentage of live/dead cells [9, 10]:

$${\rm{Total}}\;{\rm{Cell}}\;{\rm{Number = }}\;{\rm{Live}}\;{\rm{Cells + }}\;{\rm{Death}}\;{\rm{Cells}}$$

$${\rm{Percentage}}\;{\rm{of}}\;{\rm{Live}}\;{\rm{Cells = }}\;\left( {{\rm{Live}}\;{\rm{Cells/Total}}\;{\rm{Cell}}\;{\rm{Number}}} \right){\rm{*100}}$$

$${\rm{Percentage}}\;{\rm{of}}\;{\rm{Dead}}\;{\rm{Cells = }}\;\left( {{\rm{Dead}}\;{\rm{Cells/Total}}\;{\rm{Cell}}\;{\rm{Number}}} \right){\rm{*100}}$$

## Quantitative evaluation of antibiofilm effect of sub-inhibitory concentration of purified α-amylase enzyme on the quorum-sensing encoding genes in***E. coli*by qRT-PCR**

### RNA isolation, cDNA synthesis and qRT-PCR

This work was carried out to evaluate the effect of α-amylase enzyme on *fimH* and *papC* genes of *E. coli* after treatment with a sub-MIC (½ MIC) concentration of test enzymes. Biofilms grown without enzymes were selected as real-time positive controls. Briefly, the test isolate was grown overnight in LB broth medium and then sub-cultured into fresh broth. Cell cultures were diluted to give a final inoculum of approximately 2 × 10 CFU/ml in a volume of 1.5 ml. Test samples were treated with ½ MIC of purified α-amylase enzyme and the negative control was a cell suspension without treatment. After incubation at 37 °C for 6 h, total RNA was extracted from the cells using an RNA extraction kit (Applied Biosystems, USA) according to the manufacturer’s instructions. The recovered RNA was treated with RNase-free DNase for 10 min. To quantify the amount and purity of total RNA, the absorbance ratio at 260/280 nm was measured using a NanoDrop spectrophotometer (Thermo Scientific, Waltham, MA). Extracted RNA was stored at -70 °C for further experiments. For cDNA synthesis, purified RNA was reverse transcribed using a commercial cDNA synthesis kit according to the manufacturer’s instructions, and the cDNA molecules were stored at − 70 °C to be used as a template in real-time qRT-PCR reactions. For housekeeping gene, *RecA* was used as an internal control to normalize expression levels between samples. Primers for the biofilm-encoding genes *fimH* and *papC* (Macrogen labs, Korea) were used. Reactions were performed using SYBR Green/ROX qPCR Master Mix (Applied Biotech, USA). Reactions were performed in triplicate using 96-well plates. All reaction mixtures contained 3 µl cDNA, 12.5 µl SYBR^®^ Green Realtime PCR Master Mix (Applied Biosystems, USA), and 1 µl each of the forward and reverse primers listed in the table. The reaction volume was adjusted to 25 µl with 1.2 and 7.5 µl of sterile double-distilled, RNase-DNase-free water and real-time PCR amplification was performed using a thermal cycler (Qiagen Model Rotor-Gene Q 5 Plex, Germany). An initial denaturation step of 3 min at 95 °C was followed by 40 cycles comprising three steps: denaturation at 95 °C for 15 s, annealing at 60 °C for 30 s, and extension at 72 °C for 30 s. Relative gene expression data analysis was calculated as fold change using the 2-ΔΔCt method. ΔCT values ​​were determined by subtracting the CT result for the target gene from the result of the endogenous control gene 28s. Fold change in target gene expression = 2^ΔΔCq, where ΔCq = Cq [target gene] − Cq [reference gene] and ΔΔCq = ΔCq [test] − ΔCq [calibrator]. The efficiency was assumed to be the same for all samples, and RecA gene primers were chosen as reference controls to normalize the results. Each experiment was performed with three different biological replicates. For each biological sample, qPCR runs were performed in four technical replicates. Primers utilized in this study and reaction components were illustrated in Tables (1–2) [7, 12].

**Table 1:** **Primers sequence utilized in this study**

Gene name	Primer sequence
*recA* (Housekeeping gene)	Forward: CGCATTCGCTTTACCCTGACC Reverse: AGCGTGAAGGTAAAACCTGTG
*fimH* gene	Forward: TGCAGAACGGATAAGCCGTGG Reverse: GCAGTCACCTGCCCTCCGGTA
*papC* gene	Forward: TGATATCACGCAGTCAGTAGC Reverse: CCGGCCATATTCACATAAC

**Table 2:** Volumes and concentrations of qRT-PCR reaction mixture

Components	Volume (µl)
cDNA template	3
2x Maxima SYBR Green qPCR Master Mix	12.5
Forward primer	1
Reverse primer	1
Water, nuclease-free	7.5
Total	25

### Statistical analysis

The experiments were conducted three times to ensure accuracy and reliability. The statistical analyses were conducted using GraphPad Prism software (version 5). Statistical differences between groups were evaluated using one-way analysis of variance (ANOVA). Also, an unpaired t-test was used to test the significant differences between the two groups. Results were deemed significant when the *p*-value was less than 0.05.

## Results

### Biochemical identification of clinical isolates of***E. coli***

The VITEK^®^ 2 system software version 7.01 was used to identify 25 clinical isolates of *E. coli*. In Table 3, these isolates shared 96% of the traits exhibited by *E. coli* as detected by the ATCC 8729 reference strain and the standard GN (Gram-negative) card. There was a 98% confidence interval, a 3.98% standard deviation, and a 0.16% variance when 47 biochemical tests were conducted utilizing GN-ID cards. A probability value below the deviation range was observed in one of the twenty-five *E. coli* isolates.

**Table 3:** Biochemical identification of uropathogenic *E. Coli* clinical isolates using Vitek 2 systems, (+) for positive results and (-) for negative results

Test name	Result	Test name	Result	Test name	Result	Test name	Result	Test name	Result
APPA	-	ADO	-	PyrA	-	IARL	-	dCEL	-
BGAL	+	H_2_S	-	BNAG	-	AGLTp	-	dGLU	+
GGT	-	OFF	+	BGLU	+	dMAL	+	dMAN	+
dMNE	+	BXYL	-	BAIap	-	ProA	-	LIP	-
PLE	-	TyrA	+	URE	-	dSOR	+	SAC	+
dTAG	-	dTRE	+	CIT	-	MNT	-	5KG	+
ILATK	+	AGLU	-	SUCT	+	NAGA	-	AGAL	+
PHOS	-	GlyA	-	ODC	+	LDC	+	IHISa	-
CMT	+	BGUR	-	O129R	+	GGAA	-	IMLTa	-
ELLM	+	ILATa	-						

### Evaluation of antibacterial activity of purified ***B. cereus *α-amylase enzyme by determination of MIC and MBC against UPEC**

MIC and MBC levels of purified α-amylase from *B. cereus* against 25 clinical isolates of uropathogenic *E. coli* were presented in Table (4). Results of purified α-amylase were divided into two groups; the first is group A which consisted of 19 bacterial isolates (numbered 1–19) and showed results MIC and MBC of 128 µg/mL, however, group B composed of 6 bacterial isolates (numbered 20–25) recorded MIC and MBC values of 256 µg/mL and 512 µg/mL, respectively. Commercial authentic α-amylase enzyme from *B. amyloliquefaciens* was used to assess and compare the results.

**Table 4:** MIC and MBC values of purified α-amylase against UPEC test pathogens

Test	E. coli isolates group A samples ^a^	E. coli isolates group B samples ^b^
**MIC (µg/mL)**	128	256
**MBC (µg/mL)**	128	512

^a^; nineteen isolates numbered from 1 to 19

^b^; six isolates numbered from 20 to 25

### Growth curves of UPEC

**Fig. 1:**
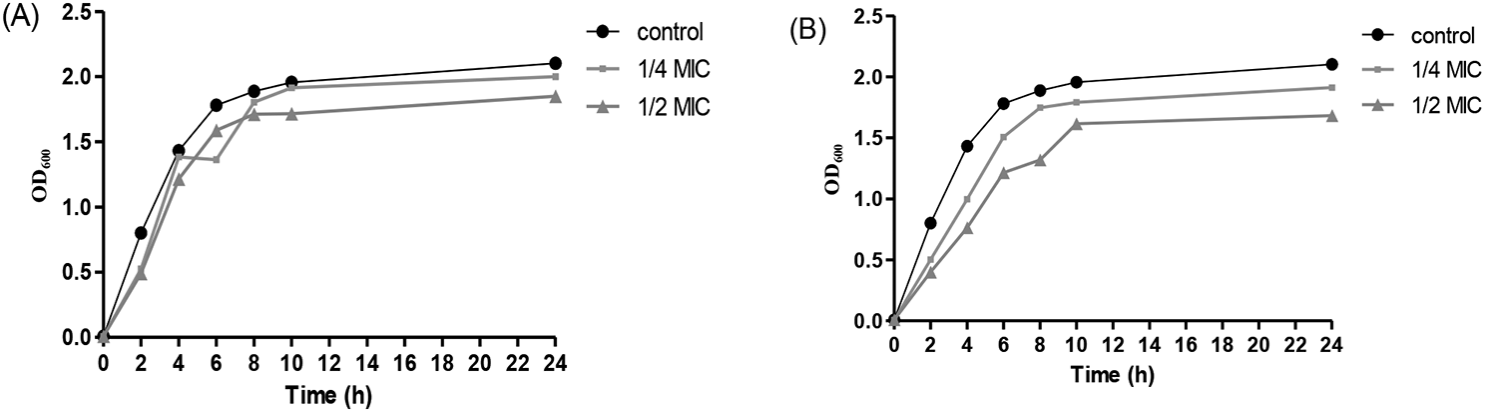
Growth curves of UPEC grown in the absence or presence of sub-MICs of (**A**) Commercial α-amylase, and (**B**) Purified α-amylase showing little but non-significant effect on the growth rate (*p* > 0.05)

Growth curves of E. coli in the absence or presence of 1/4 or 1/8 MIC of α-amylase were presented in fig. (1). As shown, there was a decrease in growth rate but without significant difference (*p* > 0.05) on the gowth of the test isolate by sub-MICs indicating the litte impact of the test enzyme as antibagrowthcterial agent.

### Determination of MBIC and MBEC of α-amylase on UPEC

The results of the crystal violet assay against 25 UPEC isolates are presented in Table 5 showed that the MBIC of purified as well as the commercial α-amylase was 128 µg/ml against uropathogenic *E. coli*. Also, the MBEC of purified α-amylase was 256 µg/ml, whereas and MBEC of the commercial enzyme was 512 µg/ml against the test isolates.

**Table 5:** The MBIC and MBEC values of purified as well as commercial α-amylases against ***E. coli isolates***

Test	Purified *B. cereus* α-amylase (µg/ml)	Commercial *B. amyloliqufaciens* α-amylase (µg/ml)
**MBIC**	128	128
**MBEC**	256	512

### Analysis of biofilm thickness and live/dead cells quantification by CLSM as well as image J software

**Fig. 2:**
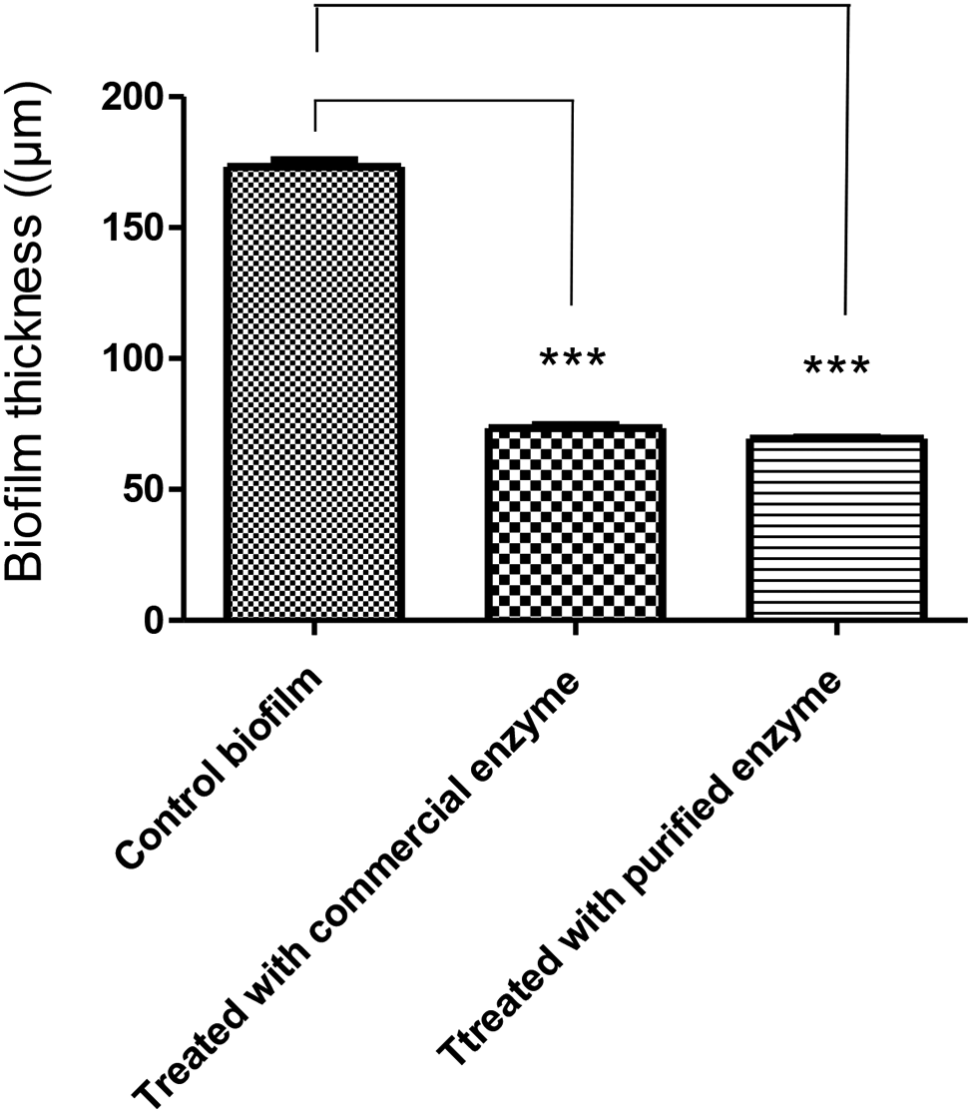
Uropathogenic *E. coli* biofilm thickness (µm) before and after treatment with commercial and purified α-amylase. *P* value < 0.0001

**Fig. 3:**
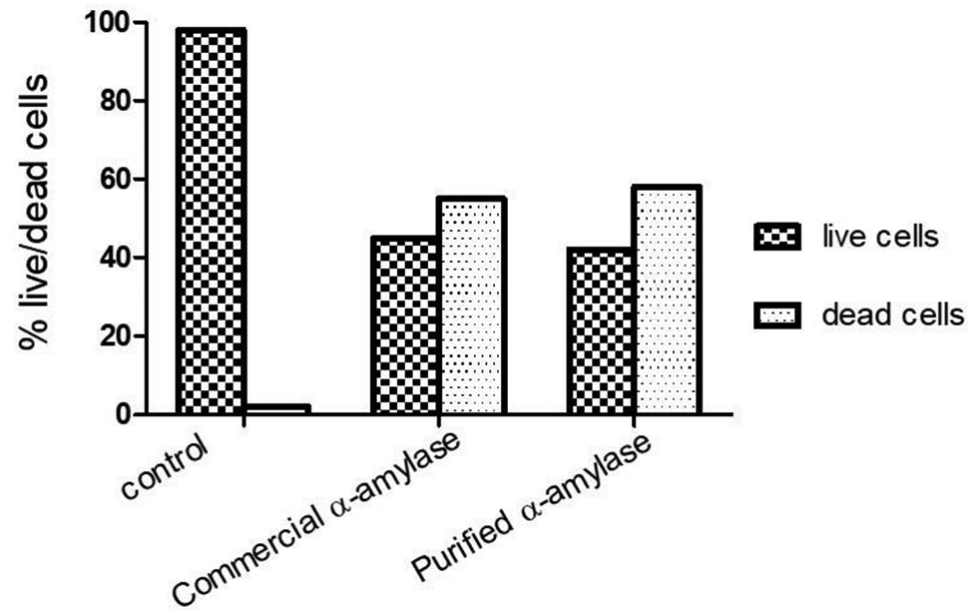
live/dead cells percentage in *E. coli* biofilms before and after treatment with commercial and purified α-amylase. *P* < 0.01

As shown in Figs. (2, 3), the biofilm thickness of *E. coli* isoltes treated with purified α-amylase was inhibited by about 55%, of which 55% were dead cells and 45% were viable cells. The biofilm thickness was significantly (*p* < 0.05) reduced from 170 μm to 75 μm. In contrast, isolates treated with commercial α-amylase exhibited a 58% reduction in biofilm thickness, with 58% of cells being non-viable and 42% being viable. This treatment resulted in a decrease in biofilm thickness from 170 μm to 70 μm (*p* < 0.05).

**Fig. 4:**
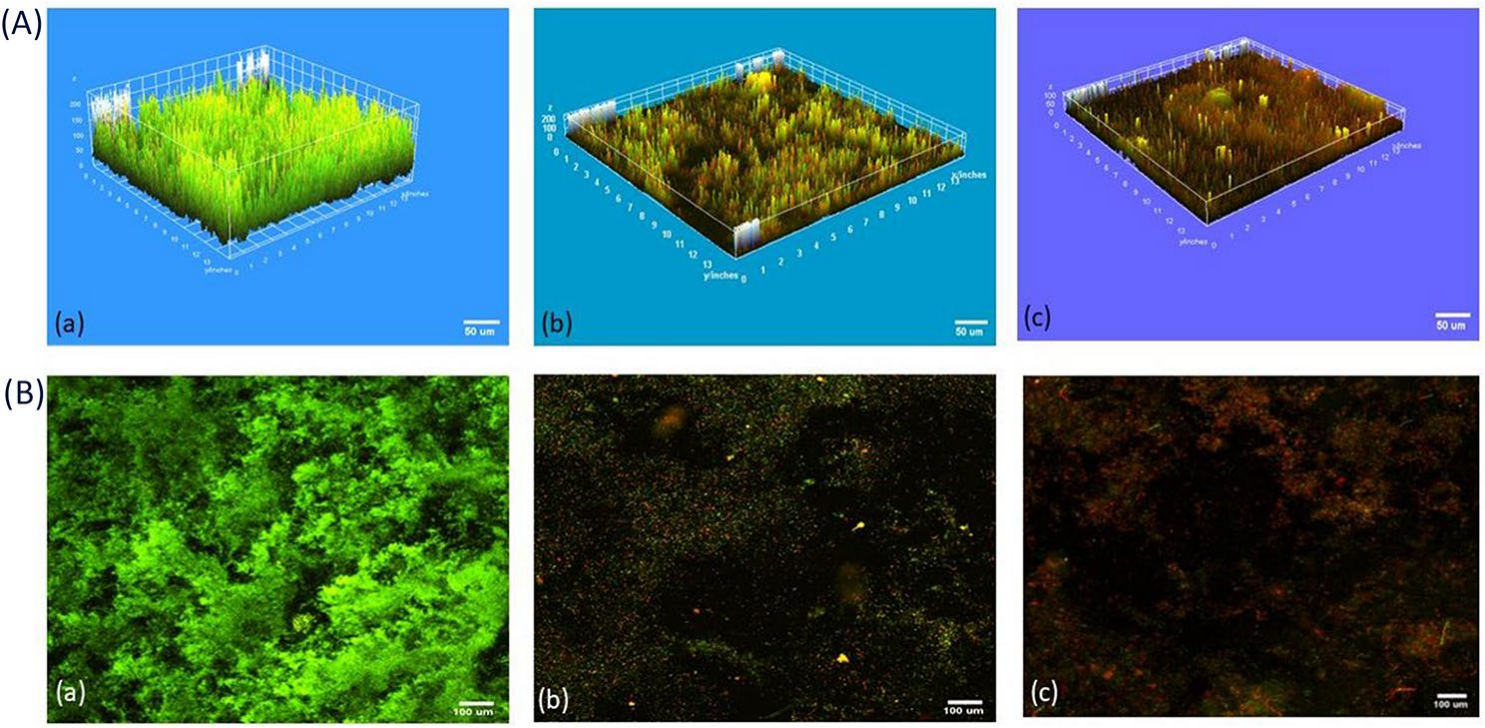
(**A**) 7 Z-axis CLSM images of *E. coli* biofilms before (**a**) and after treatment with commercial (**b**) and purified amylase (**c**). (**B**) 2D analyzed images showing the green color represents acridine-orange-stained live cells however red color represents PI-stained dead cells. Image scale is 200 μm x 200 μm. *P* value < 0.05

The results gathered demonstrated the capability of enzyme to decrease the biomass of preexisting biofilms by around 55%.The results showed that untreated *E. coli* biofilms were dense and distributed in large colonies as shown in Fig. (4). In contrast, biofilms of *E. coli* treated with α-amylase were sparse and scattered. Analysis using confocal microscopy showed that the “holes” were either not covered by cells or were covered with fragments containing DNA (PI was evenly distributed in the holes, without traces of remaining cells and oval in shape). Biofilm thickness (meaning the number of stained cells along the Z axis) was then analyzed from the time the fluorescence level became detectable until the fluorescence level met the threshold value.

**Fig. 5:**
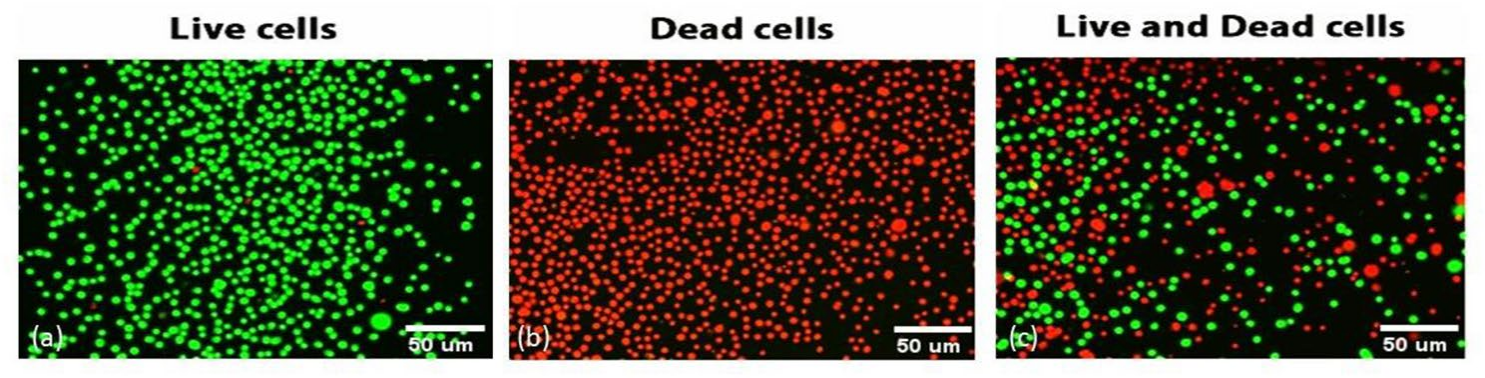
Live/dead cells pictures of RBG channels obtained from image J software showing ROI of live (green) and dead (red) particles

As shown in Fig. 5, the Otsu thresholding technique is employed to identify intensity boundaries and generate a binary mask for distinguishing live and non-live channels. A binary mask is a monochrome image in which black pixels indicate the presence of a signal, while white pixels indicate the absence of a signal. The number of cells and the percentage of area covered by cells were determined by calculating pixels as areas of interest (ROIs) based on the binary mask. The area % was determined by computing the average size of the region of interest (ROI). As a result of the staining, the size of the region of interest (ROI) ranged from substantial pieces to separate particles. Quantitative measurement of a single slice with a diameter of 9.9 μm. The number of dead cells (red stain) greatly exceeds the number of live cells (green stain). Regarding the extent of surface covered, deceased bacteria occupied 55% of the specified 200 μm x 200 μm region, but living bacteria only occupied 45% of the specified region.

### Quantitative determination of sub-MIC of purified α-amylase enzyme on the expression of some quorum sensing-encoding genes in UPEC by qRT-PCR

**Fig. 6:**
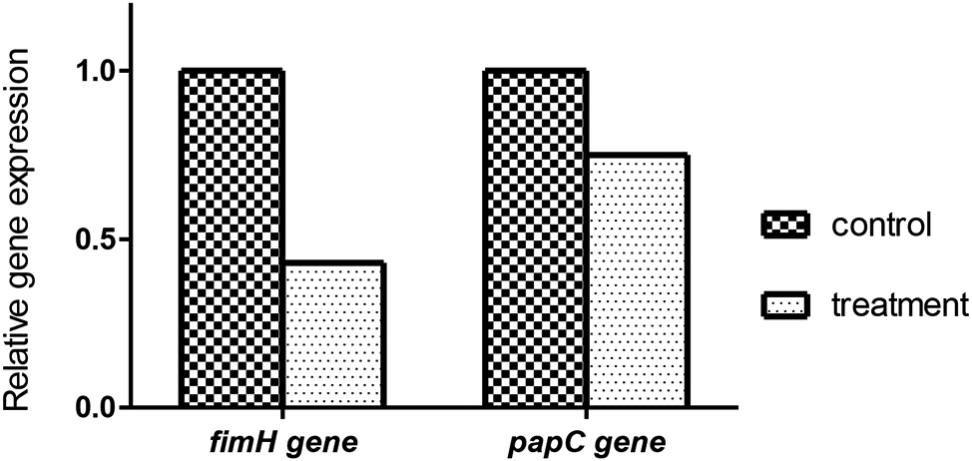
Relative expressions in fold change of *fimH* and *papC* genes after treatment with sub-MIC concentration of alpha amylase enzyme. *P* < 0.05

The test strain was pre-treated with 64 µg/mL of the purified α-amylase which corresponds to 1/2-MIC level in 76% of the test isolates. Fig 5 showed that treatment with 1/2-MIC of the test enzyme reduced the expression of *papC* (by 25%) which represented the virulence and biofilm encoding genes, and *fimH* was markedly reduced by 57% as presented in Fig. 6.

## Discussion

Many pathogens, including uropathogenic *E. coli*, have developed drug resistance as a result of the widespread misuse of antibiotics in the treatment of infections. It has been suggested that the rising rate of antibiotic resistance calls for more investigation into the development of new agents that target pathogen physiology rather than pathogen growth. Additionally, it has been demonstrated that by preventing the production of biofilms and virulence factors, the sub-MIC of certain antibiotics as well as natural compounds such as bacterial essential oils and some microbial enzymes could diminish the pathogenicity of *E. coli* pathogens [1, 13, 14]. Particularly, microbial enzymes have been proven to be effective for the degradation of the EPS of the biofilms through weakening the proteins, carbohydrates, and lipids making up the structures of the EPS [2]. The goal of the current investigation was to find out if sub-MIC levels of our purified microbial α-amylase could influence the virulence genes of *E. coli via* preventing the production of biofilms or even eradicate the pre-established biofilm.

In vitro investigations have shown that several enzymes have antibiofilm activities. It has been demonstrated that the *B. subtilis* α-amylase affected the biofilm topologies (44–62%) of clinical isolates including MRSA, *Vibrio cholerae*, and *P. aeruginosa*, as well as the biofilm formation process [15]. Furthermore, it was observed that commercially available α-amylases (100 mg/mL) displayed antibiofilm action (> 82%), demonstrating inhibition of *S. aureus* biofilm formation [16]. Furthermore, Watters et al. [17] created *S. aureus* biofilm model (6 bacterial strains) by adding plasma to the growth media, which was intended to replicate the wound-like environment. Enzymes like α-amylase, lysostaphin, bromelain, and papain were then added to the growth media to test the enzymes’ ability to prevent biofilm formation in vitro. It was found that *S. aureus* produced a strong biofilm when 10% plasma was present. However, biofilm reduction of approximately 17–92%, 47–96%, 88–95%, and 68–94% was noted in the treatments with α-amylase (10 mg/mL), lysostaphin (50 µg/mL), bromelain (100 µg/mL), and papain (100 µg/mL), respectively [17]. Our work showed that treating UPEC with 256 µg/mL (MBEC) of the purified α-amylase resulted in 98% reduction in the biofilm whereas higher concentration of the commercial α-amylase is required to get the same percentage of biofilm reduction. Vaikundamoorthy et al. [18] reported that α-amylase derived from marine sources exhibited remarkable antibiofilm action against *S. aureus* and *P. aeruginosa*. Additionally, it has been observed that the biofilm structure from *P. aeruginosa*, *B. subtilis*, and *S. aureus* can be broken down by 75–82% using protease (10 U/mg), amylase (8 U/mg), and pectinase (10.086 U/mg) that are created through solid-state fermentation from a fungus species called *Aspergillus clavatus* [19]. *Penicillium janthinellum’s* α-amylase exhibited over 80% antibiofilm activity against *Salmonella* enterica, *P. aeruginosa*,* S. aureus*, and *E. coli* [20].

In this work, we investigated the direct effect of the purified α-amylase on planktonic UPEC isolates showing MIC and MBC ranged between 128 and 512 µg/mL denoting direct antibacterial impact. Moreover, there was also an inhibitory (MBIC 128 µg/mL) as well as an eradication effect (MBEC; 128 µg/mL) on the biofilm forming UPEC. Interestingly, the thickness of the biofilm was reduced (56%) after treatment with 256 µg/mL of the test enzyme indicating its degradation effect on the EPS forming the matrix of the biofilm. The mechanism by which enzymes destroy the physical integrity of EPS is a degradation process that weakens the proteins, carbohydrates, and lipids that make up the EPS structure [15, 21]. When α-amylase compounds originating from human, fungal, and bacterial sources were incubated with *E. coli* biofilms, there was a discernible decrease in the accumulation of biofilms [22]. On the other hand, there was a decreased inhibitory impact observed for β-amylase. Since α-amylase can act simultaneously anywhere on the substrate, it tends to act faster than β-amylase. This difference in biofilm reduction capability might be due to the fact that β-amylase is an exo-acting carbohydrolase that hydrolyzes the α-1,4-glucosidic linkages of starch only from the non-reducing end of the polysaccharide. Apart from its ability to prevent the development of *E. coli* biofilms, α-amylase could also quickly break down pre-existing biofilms at concentrations as low as 10 mg/ml. However, longer incubation times were needed to attain a similar level of biofilm reduction at an enzyme concentration of 1 mg/ml. Similar to what was shown for biofilm inhibition, the capacity to reduce biofilm was consistent across all surfaces and isolates examined, as well as with α-amylase compounds sourced from *B. subtilis* and *A. oryzae* [23].

Although many previous studies have described the effects of natural substances such as acids and essential oils on *E. coli* quorum sensing genes, studies done regarding the effects of the bacterial enzyme α-amylase on the expression of Gram-negative bacterial biofilms are very limited. Closely related to this activity, a very limited study provides evidence that the QS mechanism in Gram-negative bacteria such as *E. coli* involved special molecules called acylhomoserine lactones (AHLs). These molecules contain a lactone ring that plays an important role in QS signaling and biofilm formation, some extracellular enzymes such as lipases, proteases, and amylases cleave this lactone ring in pathogenic bacterial cells and interfere with the expression of QSM and coding genes [7, 24, 25]. In the present work, purified α-amylase reduced the expression of both genes; *papC* (25%) and *fimH* (57%) which represented virulence as well as biofilm encoding genes where 90% of UPEC showed adhesion using type I fimbriae. In fact, the antibiofilm activity of bacterial α-amylase enzyme is originated from its ability to degrade the EPS which represent 75% of biofilm matrix by breaking out the α-1,4- glycosidic bonds in EPS to small sugar units, then the entrapped M.O is now exposed to external immunity, so α-amylase has a well-known antibiofilm and moderate killing activity on *E. coli* cells according to CLSM results. Also, α-amylase enzyme is considered as one of glycoside hydrolase enzymes that revealing effectiveness against quorum sensing N-AHLs, but the exact mechanism is still unknown. in this work we clarified the effect of α-amylase enzyme on biofilm encoding genes because a few researches revealed that the extracellular hydrolytic enzymes like acyclases and lactonases has a promising inhibitory effect on N-AHLs during bacterial biofilm production cascade by blocking the auto inducer signaling and binding to their receptors and as a result interfering with transcription and release of biofilm encoding genes in bacterial cells. In contrast, Hangler et al. [26] observed that the serine protease Esperase HPF (subtilisin) as one of important hydrolytic enzymes affected both the attachment and the detachment of a multispecies biofilm, suggesting that, in this case, the enzyme effectively degraded both protein-based adhesives and proteins contained in the matrix. Recent work also showed that differences in the chemical composition of the EPS are reflected in the vulnerability of biofilms to enzymatic treatments. Charu Goel et al. [27] revealed that some hydrolytic enzymes like amylases and cellulases causes efficient degradation of biofilm matrix or EPS of Gram-negative bacteria like *Pantoea agglomerans*. So, this work demonstrates the biofilm inhibitory activity of α-amylase enzyme on phenotypic as well as genotypic levels.

In summary, uropathogenic *E. coli* biofilm was successfully inhibited by purified α-amylase derived from a microbial source, *Bacillus cereus*. Confocal laser scanning fluorescence microscopy revealed that the purified α-amylase significantly inhibited EPS synthesis by reducing the thickness and decreasing the number of viable cells. Furthermore, it induced downregulation in the expression of some important quorum-sensing genes in *E. coli*. α-amylase also demonstrated good antibacterial activity against clinical isolates of uropathogenic *E. coli*. Our results suggest that amylase, a natural and safe substance, has significant potential in the healthcare system, providing new insights into *E. coli* biofilm fouling and the inhibition and termination of *E. coli* biofilm formation in clinical medicine.

## Data Availability

No datasets were generated or analysed during the current study.

## References

[1] Guofeng Dong J, Li et al. Effects of sub-minimum inhibitory concentrations of ciprofloxacin on biofilm formation and virulence factors of Escherichia coli. The Brazilian Journal of Infectious Diseases. Volume 23, Issue 1, January–February. 2019, Pages 15–21. 2019. 10.1016/j.bjid.2019.01.004 PMC9428002 30796889

[2] Mohammed T, Mahmood BA, Abdullah. The relationship between biofilm formation and presence of fimH and mrkD genes among *E. Coli* and *K. pneumoniae* isolated from patients in Mosul. Mosul Nurs J, 1, 3, 2015.

[3] Hojjatolah Zamani A, Salehzadeh. Biofilm formation in uropathogenic Escherichia coli: association with adhesion factor genes. Turk J Med Sci. 2018;48:162–7. 29479978 10.3906/sag-1707-3

[4] Daira Melendez. Whole genome analysis of extraintestinal pathogenic Escherichia coli (ExPEC) isolated from the endangered Southern Resident Killer whales (SRKW; Orcinus orca). University of Washington; 2019. 10.1093/jac/dkz159 31032855

[5] Susan M, Cotterill, et al. recA protein from Escherichia coli. A very rapid and simple purification procedure: binding of adenosine 5’-triphosphate and adenosine 5’-diphosphate by the homogeneous protein. Biochemistry. 2000;21:18, 4332–7. 10.1021/bi00261a023 6751387

[6] Robert W, Maul et al. Roles of the Escherichia coli RecA protein and the Global SOS response in effecting DNA polymerase selection in vivo. J Bacteriol, vol,187, 22, 2005. 10.1128/JB.187.22.7607-7618.2005 PMC1280315 16267285

[7] Shlomit, Dor. Dov Prusky, and Livnat Afriat-Jurnou. Bacterial quorum-quenching lactonase hydrolyzes fungal mycotoxin and reduces pathogenicity of Penicillium Expansum—suggesting a mechanism of bacterial antagonism. Journal of fungi; 2021. 10.3390/jof7100826 PMC8537011 34682247

[8] Amal M, AboKamer IS, AbdElsalam, et al. A promising microbial αamylase production, and purifcation from *Bacillus cereus* and its assessment as antibioflm agent against Pseudomonas aeruginosa pathogen. Microb Cell Fact. 2023;22:141. 37528448 10.1186/s12934-023-02139-6 PMC10391895

[9] Ali saadi Al-Baer and, Asmaa A, Hussein. Isolation and Identification of Escherichia coli ProducingCytosine Deaminase from Iraqi patients, International Journal of Advanced Research in Biological Sciences ISSN: 2348–8069. (2017). 4(11): 1-6Lorenzo Drago, How to Study Biofilms after Microbial Colonization of Materials Used in Orthopaedic Implants. Int. J. Mol. Sci. 2016, 17(3), 293. 10.3390/ijms17030293 PMC4813157 26927075

[10] Clinical and Labortaory standard institute. Performance standard for antimicrobial susceptibility testing. 30th edition. CLSI supplement M100 Wayne PA; Clinical and Labortaory standard institute.

[11] Milos, Legner, et al. Evaluating the effects of disinfectants on bacterial biofilms using a Microfluidics Flow Cell and Time-Lapse fluorescence Microscopy. Microorganism MDPI. 2020;8(11):1837. 10.3390/microorganisms8111837. 10.3390/microorganisms8111837 PMC7700140 33266442

[12] Lee SY, Lee YS. Susceptibility of oral streptococci to Chlorhexidine and Cetylpyridinium Chloride. Biocontrol Sci. 2019;24(1):13–21. 30880309 10.4265/bio.24.13

[13] Amal M, Abo Kamer AA, Abdelaziz, Ahmed M, Nosair LA, Al-Madboly. (2022). Characterization of newly isolated bacteriophage to control multi-drug resistant Pseudomonas aeruginosa colonizing incision wounds in a rat model: in vitro and in vivo approach. Life Sciences 310 (2022) 121085. 10.1016/j.lfs.2022.121085 36265569

[14] Lamiaa Al-Madboly. (2022). A novel triple combination to combat serious infections with carbapenem-resistant Acinetobacter baumannii in a mouse Pneumonia Model. 10.1128/ Microbiology Spectrum.02710-21. 10.1128/spectrum.02710-21 PMC9603289 35975993

[15] Kalpana BJ, Aarthy S, Pandian SK. Antibiofilm activity of α-amylase from Bacillus subtilis S8-18 against biofilm forming human bacterial pathogens. Appl Biochem Biotechnol. 2012;167(6):1778–94. 10.1007/s12010-011-9526-2. Epub 2012 Feb 21. PMID: 22350933. 22350933 10.1007/s12010-011-9526-2

[16] Craigen B, Dashiff A, Kadouri DE. (2011). The use of commercially available alpha-amylase compounds to inhibit and remove *Staphylococcus aureus* biofilms. *Open Microbiol. J.* 5 21. 10.2174/1874285801105010021 10.2174/1874285801105010021 PMC3134978 21760865

[17] Watters CM, Burton T, Kirui DK, Millenbaugh NJ. Enzymatic degradation of in Vitro Staphylococcus Aureus Biofilms Supplemented with Human plasma. Infect. Drug Resist. 2016;9:71–8. 10.2147/IDR.S103101. 10.2147/IDR.S103101 PMC4854256 27175088

[18] Ramalingam V, Rajaram R, Archunan G, Padmanabhan P, Gulyás B. Structural characterization, Antimicrobial, Antibiofilm, antioxidant, Anticancer and Acute Toxicity properties of N-(2-hydroxyphenyl)-2-phenazinamine from Nocardiopsis exhalans (KP149558). Front Cell Infect Microbiol. 2022;12:794338. 10.3389/fcimb.2022.794338. PMID: 35663469; PMCID: PMC9161293. 35663469 10.3389/fcimb.2022.794338 PMC9161293

[19] Singh AK, Mukhopadhyay M. Overview of fungal lipase: a review. Appl Biochem Biotechnol. 2012;166(2):486–520. 22072143 10.1007/s12010-011-9444-3

[20] Nagraj AK, Gokhale D. Bacterial biofilm degradation using extracellular enzymes produced by *Penicillium Janthinellum* EU2D-21 under submerged fermentation. Adv Microbiol. 2018;8:687–98. 10.4236/aim.2018.89046.

[21] Sayem SMA, Chowdhury AJMT, Alam MZ, Sarker PK. Antibiofilm Activity of Crude Cell Free Extract from *Bacillus subtilis* S01 against E. Coli. J Sci Res. 2018;10(2):211–21.

[22] Goel C, Shakir C, Tesfaye A, Sabu KR. Antibiofilm potential of alpha-amylase from a Marine Bacterium, Pantoea agglomerans. Can J Infect Dis Med Microbiol Article ID. 2022;7480382:10. 10.1155/2022/7480382. 10.1155/2022/7480382 PMC9033359 35462682

[23] Lahiri D, Nag M, Banerjee R, Mukherjee D, Garai S, Sarkar T, Dey A, Sheikh HI, Pathak SK, Edinur HA, Pati S, Ray RR. Amylases: Biofilm Inducer or Biofilm inhibitor? Front. Cell Infect Microbiol. 2021;11:660048. 10.3389/fcimb.2021.660048. 10.3389/fcimb.2021.660048 PMC8112260 33987107

[24] Whelan S, Lucey B, Finn K. (2023). Uropathogenic Escherichia coli (UPEC)-Associated Urinary Tract Infections: The Molecular Basis for Challenges to Effective Treatment. Microorganisms. 2023; 11(9): 2169. 10.3390/microorganisms11092169 10.3390/microorganisms11092169 PMC10537683 37764013

[25] Jiamu K, Qianqian, Li, Liu, et al. The specific effect of gallic acid on Escherichia coli biofilm formation by regulating pgaABCD genes expression. Appl Microbiol Biotechnol. 2018;102:1837–46. 29327068 10.1007/s00253-017-8709-3

[26] Hangler M, Burmølle M, Schneider I, Allermann K, Jensen B. The serine protease Esperase HPF inhibits the formation of multispecies biofilm. Biofouling. 2009;25(7):667 – 74. doi: 10.1080/08927010903096008. PMID: 20183125. 10.1080/08927010903096008 20183125

[27] Goel C, Shakir C, Tesfaye A, Raghavanpillai Sabu K, Idhayadhulla A, Manilal A, Woldemariam M, Vijayan N, Shah S. Antibiofilm potential of alpha-amylase from a Marine Bacterium, Pantoea agglomerans. Can J Infect Dis Med Microbiol. 2022;2022:7480382. 10.1155/2022/7480382. PMID: 35462682; PMCID: PMC9033359. 35462682 10.1155/2022/7480382 PMC9033359

